# Apigenin targets fetuin-A to ameliorate obesity-induced insulin resistance

**DOI:** 10.7150/ijbs.91695

**Published:** 2024-02-11

**Authors:** Man-Chen Hsu, Chia-Hui Chen, Mu-Chun Wang, Wen-Hua Chen, Po-An Hu, Bei-Chia Guo, Ru-Wen Chang, Chih-Hsien Wang, Tzong-Shyuan Lee

**Affiliations:** 1 Graduate Institute and Department of Physiology, College of Medicine, National Taiwan University, Taipei, Taiwan.; 2 Department of Cardiovascular Surgery, Min-Sheng General Hospital, Taoyuan, Taiwan.; 3 Cardiovascular Surgery, Department of Surgery, National Taiwan University Hospital and College of Medicine, Taipei 10051, Taiwan.

**Keywords:** apigenin, fetuin-A, CK2α, insulin receptor, insulin resistance

## Abstract

Fetuin-A, a hepatokine secreted by hepatocytes, binds to insulin receptors and consequently impairs the activation of the insulin signaling pathway, leading to insulin resistance. Apigenin, a flavonoid isolated from plants, has beneficial effects on insulin resistance; however, its regulatory mechanisms are not fully understood. In the present study, we investigated the molecular mechanisms underlying the protective effects of apigenin on insulin resistance. In Huh7 cells, treatment with apigenin decreased the mRNA expression of fetuin-A by decreasing reactive oxygen species-mediated casein kinase 2α (CK2α)-nuclear factor kappa-light-chain-enhancer of activated B activation; besides, apigenin decreased the levels of CK2α-dependent fetuin-A phosphorylation and thus promoted fetuin-A degradation through the autophagic pathway, resulting in a decrease in the protein levels of fetuin-A. Moreover, apigenin prevented the formation of the fetuin-A-insulin receptor (IR) complex and thereby rescued the PA-induced impairment of the insulin signaling pathway, as evidenced by increased phosphorylation of IR substrate-1 and Akt, and translocation of glucose transporter 2 from the cytosol to the plasma membrane. Similar results were observed in the liver of HFD-fed mice treated with apigenin. Collectively, our findings revealed that apigenin ameliorates obesity-induced insulin resistance in the liver by targeting fetuin-A.

## 1. Introduction

Fetuin-A, also known as α2-Heremans-Schmid-glycoprotein (AHSG), is a glycoprotein synthesized mainly by hepatocytes and secreted into the bloodstream to exert metabolic actions; therefore, it is considered a hepatokine 1. Fetuin-A is a multifunctional protein in humans that plays an important role in regulating various physiological functions, including osteogenesis, systemic mineral metabolism, inflammation, energy homeostasis, lipid metabolism, cell growth, and survival 2-6. Several lines of evidence suggest that fetuin-A, as a chaperone, inhibits systemic calcification and prevents coronary artery disease and kidney stone formation 7-8. However, growing evidence indicates that elevated circulating fetuin-A levels are strongly associated with insulin resistance 9. Fetuin-A, an endogenous inhibitor of tyrosine kinases, binds to the extracellular domain of insulin receptors (IRs) and impedes the activation of IR signaling pathway by inhibiting IR autophosphorylation, resulting in decreased insulin sensitivity and glucose utilization, ultimately increasing the risk of type 2 diabetes 10-12. In addition to the nuclear factor kappa-light-chain-enhancer of activated B cells (NF-κB)-mediated transcriptional regulation, post-translational modifications such casein kinase 2α (CK2α)-dependent phosphorylation at Ser120 and Ser312 of fetuin-A is a key molecular mechanism for regulating the protein stability of fetuin A 13-14. Ren et al. reported that the phosphorylation status of fetuin-A (p-fetuin-A) is highly associated with obesity and insulin resistance, and plays a critical role in inhibiting the insulin signaling pathway 15. However, little is known regarding therapeutic strategies targeting fetuin-A for the treatment of obesity-induced insulin resistance. To this end, further investigations are warranted to develop potential drugs that inhibit fetuin-A signaling.

Apigenin belongs to the flavone subclass of flavonoids and is ubiquitous in many vegetables and fruits, particularly in parsley, oranges, and onions 16-17. The beneficial effects of apigenin on human health have been extensively studied owing to its various biological activities, including antioxidant, anti-inflammatory, and anti-obetisy effects 18-20. The pharmacokinetics of apigenin in rats were determined, and it was found that after a single dose of 10 mg of apigenin by oral administration for 10 days, undegraded apigenin entered the circulation through intestinal absorption 21. Apigenin was detectable at 9 h post-administration and increased to reach a maximum of ~0.42 mg/mL at 24 h post-ingestion, which then slowly declined to an undetectable level at ~250 h post-administration 21. Recently, we showed that apigenin prevents hepatic lipid accumulation by activating the autophagy pathway 22. Additionally, apigenin has been reported to reduce oxidative stress in the liver by decreasing the lipid peroxidation product 4-hydroxy-2-nonenal (4-HNE) and increasing the activity of antioxidant enzymes, including heme oxygenase-1 (HO-1), glutathione peroxidase (GPx), and superoxide dismutase (SOD) 23-25. Jung et al. demonstrated that apigenin ameliorated insulin resistance in the livers of high-fat diet (HFD)-fed mice 26. Although the protective effects of apigenin for obesity-mediated metabolic disorders have been established, the molecular mechanisms underlying the beneficial effects of apigenin on obesity-induced insulin resistance are yet to be elucidated. Whether apigenin improves the deregulation of the insulin signaling pathway mediated by the inhibition of fetuin-A function warrants further investigation.

Given the beneficial effects of apigenin on human diseases, we aimed to investigate the effects of apigenin and its molecular mechanism of action on palmitic acid (PA)-induced and HFD-induced insulin resistance *in vitro* and* in vivo*. First, we investigated the effects of apigenin on fetuin-A expression. Next, we investigated the molecular mechanisms underlying the protective effects of apigenin on fetuin-A-mediated deregulation of insulin signaling. Our findings suggest that treatment with apigenin decreases the protein levels of fetuin-A and p-fetuin-A, prevents the formation of the fetuin-A-IR complex, and improves glucose utilization, consequently attenuating the insulin resistance induced by PA or obesity.

## 2. Materials and methods

### 2.1. Reagents

Apigenin, palmitate (PA), bafilomycin A1 (BafA1), 3-(4,5-Dimethylthiazol-2-yl)-2,5-diphenyltetrazolium bromide (MTT), chloroquine (CQ), 3-methyladenine (3-MA), cycloheximide (CHX), and 2-deoxy-2-[(7-nitro-2,1,3-benzoxadiazol-4-yl) amino]-D-glucose (2-NBDG) were from Cayman Chemical (Montgomery, TX, USA). Rabbit antibodies for fetuin-A (A305-236A) was from Bethyl Laboratories (Ann Arbor, MI, USA). Rabbit antibody for casein kinase 2α (CK2α, #2656S), phosphor-serine (#9631), microtubule-associated protein 1A/1B-light chain 3 (LC3, #4108), sequestosome 1 (p62, #5114), insulin receptor (#74118S), adipose triglyceride lipase (ATGL, #2138), and hormone-sensitive lipase (HSL, #4107) were from Cell Signaling Technology (Beverly, MA, USA). Protein A/G PLUS-agarose beads (sc-2003) and rabbit antibodies for phosphor-insulin receptor substrate-1 at Tyr-989 (p-IRS-1, sc-17200), IRS (sc-559), protein kinase B (Akt) (sc-1619) and mouse antibody for insulin receptor (sc-57344) were obtained from Santa Cruz Biotechnology (Santa Cruz, CA, USA). Rabbit antibodies for glucose transporter 2 (GLUT2, A12307), NF-κB (A19653), phosphor-NF-κB at S-311 (p-NF-κB, AP0445), phosphor-Akt at S473 (AP0140), and nuclear factor erythroid 2-related factor 2 (Nrf2, A1244) were purchased from ABclonal Science (Woburn, MA, USA). Rabbit antibodies for 4-HNE (ab46545), SOD1 (ab13498), and SOD2 (ab13533); rat antibody for F4/80 (ab6640); mouse fetuin-A ELISA kit (AB205074) were obtained from Abcam (Cambridge, MA, USA). Mouse antibody for GPx (AF3798), mouse tumor necrosis factor-alpha (TNFα, MTA00), and interleukin 6 (IL-6, M6000B-1), IL-1β (MLB00C-1), IL-4 (M4000B-1) ELISA kit were obtained from R&D systems (Minneapolis, MN, USA). Rabbit antibody for HO-1 (ADI-SPA-895) was obtained from Enzo Life Sciences (Stressgen, Victoria, BC, Canada). Rabbit antibody for lysosomal acid lipase (LAL, GTx101169) was obtained from GeneTex (Irvine, CA, USA). Mouse antibody for β-actin and rabbit antibody myeloperoxidase (MPO, 22225-1-AP) were obtained from Proteintech (Rosemont, IL, USA). Dihydroethidine (DHE) and 2′,7′-dichlorofluorescin diacetate (DCFH-DA) were obtained from Molecular Probes (Eugene, OR, USA). N-acetylcysteine (NAC), apocynin (APO), 2-[(4-bromophenyl) methylene]-N-(2,6-dimethylphenyl)-hydrazinecarboxamide] (EGA), malondialdehyde (MDA) assay kit, and proximity ligation assay (PLA) kit, duolink* in situ* detection reagents green (DUO92014-30RXN) were obtained from Sigma-Aldrich (St Louis, MO, USA). Cholesterol, high-density lipoprotein (HDL)-cholesterol (HDL-c), and triglyceride assay kits were purchased from Randox (Crumlin, Antrim, UK).

### 2.2. Cell culture

Human hepatoma cell line Huh7 cells were cultured with Dulbecco's modified Eagle's medium (DMEM) (Thermo Fisher Scientific, Waltham, MA) supplemented with 10% fetal bovine serum (FBS) (Thermo Fisher Scientific) and 100 (U/mL) penicillin / 100 (µg/mL) streptomycin (HyClone, Logan, UT, USA) in a humidified incubator at 37°C, 95% air, and 5% CO_2_.

### 2.3. Animal

This study conformed to the Guide for the Care and Use of Laboratory Animals (Institute of Laboratory Animal Resources, eighth edition, 2011), and all animal experiments were approved by the Animal Care and Utilization Committee of College of Medicine, National Taiwan University (No. 20230110). Male wild-type (WT) C57BL/6 mice at 8 weeks old were obtained from the Laboratory Animal Center of the National Taiwan University College of Medicine (Taipei, Taiwan) and were housed in barrier facilities on a 12-h light/12-h dark cycle. Mice were housed in groups of 3-4 per cage and randomly divided into two groups. Mice received oral administration with either oil (vehicle control, n=7) or apigenin (20 mg/kg/day, n=7) and were fed with a high-fat diet (HFD, 60% fat) for 12 weeks (New Brunswick, NJ, USA). At the end of the experiment, mice were euthanized with CO_2_ after measuring the body weight of mice. The livers were isolated and weighed. Plasma was isolated from citrated blood (3.2% sodium citrate, 1:10) by centrifugated for 10 min at 1500 g, 4°C, and then stored at -80°C until further analysis.

### 2.4. Quantitative real-time PCR (qRT-PCR)

Total RNA was isolated from cells by NucleoZOL (Takara Bio USA, Inc., CA, USA) and converted into cDNA use of ToolsQuant II Fast RT kit (BIOTOOLS Co., Taipei, Taiwan). cDNAs were then used as templates for qRT-PCR. qRT-PCR involved the TaqMan probe-based real-time quantification system (Foster, CA, USA). The levels of mRNA were calculated relative to GAPDH mRNA as the invariant control. The sequences of primers are as following: human fetuin-A forward primer: 5′-CACAGGTAACAGCTCCGTGA-3′; human fetuin-A reverse primer: 5′-TGATTCCGCATACCCCAGTG-3′; human GAPDH forward primer: 5′-AACGGGAAGCTTGTCATCAATGGAAA-3′; human GAPDH reverse primer: 5′-GCATCAGCAGAGGGGGCAGAG-3′.

### 2.5. Measurement of intracellular levels of reactive oxygen species (ROS)

Huh7 cells were pretreated with DHE (10 µM) or DCFH-DA (20 µM) at 37°C for 30 min. The DHE or DCFH-DA-containing cell medium was removed and replaced with a fresh medium. Intracellular ROS were observed at 0, 5, 10, 15, 30, and 60 min after treated with palmitic acid. The fluorescence intensity in cellular lysates was analyzed using fluorometry at 518 nm excitation and 605 nm emission for ethidium (ETH) and 488 nm excitation and 530 nm emission for DCF, respectively. Images were captured under a Nikon TE2000-U microscope with an image analysis system.

### 2.6. 2-NBDG Glucose uptake

Huh7 cells were incubated with serum-free medium for 2 h at 37°C and then treated with insulin (100 nM) for 15 min. Then treated cells were incubated with fluorescent hexose 50 µM 2-NBDG (a glucose analogue) for 20 min. The medium was then discarded and the cells were washed with PBS. The fluorescence intensity of cell lysates was analyzed at 485 nm excitation and 535 nm emission wavelengths by SpectraMax i3x fluorometry (San Jose, CA, USA). The images of glucose uptake were captured by Zeiss LSM 880 confocal microscope with Zen software.

### 2.7. Histology examination

The livers were fixed with 10% neutral buffered formalin, embedded in paraffin then sliced into 8 µm sections and subjected to histological examination. Cryosections were subjected to oil red O staining and the deparaffinized section was subjected to hematoxylin and eosin (H&E) staining, immunohistochemistry, and proximity ligation assay (PLA), then the images were viewed under Motic TYPE 102M microscope (Motic Images Plus 2.0, Xiamen, China).

### 2.8. Immunohistochemistry

The deparaffinized sections were incubated with retrieval buffer (10 mM sodium citrate, 0.05% Tween 20, pH 6.0) for 10 min at 37°C. The sections were blocked with 2% BSA for 60 min at 37°C and incubated with 4-HNE or GLUT2 antibody for 2 h at 37°C and then the FITC-conjugated secondary antibody for overnight at 4°C. Images were observed under Zeiss LSM 880 confocal microscope with Zen software (Carl Zeiss AG, Oberkochen, Germany).

### 2.9. Western blot analysis

The Huh7 cells or livers were lysed in immunoprecipitation lysis buffer (50 mM Tris pH7.5, 5 mM EDTA, 300 mM NaCl, 1% Triton X-100, 1 mM phenylmethylsulfonyl fluoride, 10 µg/mL leupeptin, 10 µg/mL aprotinin and phosphatase inhibitor cocktail II and III) and the total protein was extracted. Aliquots of protein (50 μg) or plasma (2 μl) mixed with 5 μl loading dye (250 mM Tris HCl pH6.8, 500 mM dithiothreitol, 10% SDS, 50% glycerol and bromophenol blue) were separated on 8%, 12% SDS gels and then transblotted onto the PVDF membrane (Millipore, Bedford, MA, USA). After being blocked with 5% skim milk, the blotting membrane was then incubated with the primary antibodies, followed by the corresponding horseradish peroxidase-conjugated secondary antibodies. The protein bands were visualized by using an enzyme-linked chemiluminescence detection kit (Perkin, Waltham, MA, USA), and the band density was measured using the quantitative software (TotalLab, Newcastle upon Tyne, UK).

### 2.10. Immunoprecipitation (co-IP)

Huh7 cells and the livers were lysed with NP-40 lysis buffer (10 mM HEPES, 10 mM KCl, 1.5 mM MgCl_2_, 0.5% nodiet P-40, 0.02% sodium azide in PBS) containing protease inhibitors (1 mM phenylmethylsulfonyl fluoride, 10 µg/mL leupeptin, 10 µg/mL aprotinin) and phosphatase inhibitor cocktail II and III. After centrifugation at 10,000 g for 5 min at 4°C, 1000 µg supernatant was incubated with anti-fetuin-A antibody overnight at 4°C with protein A/G-PLUS-Agarose. The immune complexes were precipitated by centrifugation at 1400 g for 3 min and washed 3 times with ice-cold PBS. Pellets were resuspended and boiled in SDS lysis buffer (1% Triton, 0.1% SDS, 0.2% sodium azide, 0.5% sodium deoxycholate, 1 mM PMSF, 10 µg/mL aprotinin and 10 µg/mL leupeptin) for 5 min. Samples then were heated at 95°C for 10 min and subjected to western blot analysis.

### 2.11. Plasma lipid profile analysis

Blood was collected via a cardiac puncture. After centrifugations, plasma was isolated and the levels of triglycerides, total cholesterol, HDL-c, and non-HDL-c were evaluated by using the corresponding test strips of Spotchem EZ SP 4430 (ARKRAY, Kyoto, Japan).

### 2.12. Hepatic lipid analysis

The lipids in the liver were extracted by use of n-hexane/isopropanol (3:2, v/v) for 2 h on a shaker. After drying, the extracted lipids were dissolved by using the commercial solutions, and the levels of intracellular total cholesterol, cholesteryl ester, triglycerides, free fatty acids, and glycerol were assessed by using fluorescence assay kits (BioVision, Milpitas, CA, USA). The fluorescence intensity of cell lysates was analyzed at 530 nm excitation and 590 nm emission wavelengths by SpectraMax i3x fluorometry (San Jose, CA, USA).

### 2.13. Glucose tolerance tests (OGTT) and insulin tolerance tests (ITT)

For OGTT, mice were fasted overnight, and blood glucose was measured at 0, 30, 60, 90, and 120 min after oral gavage of glucose (1.0 g/kg/of body weight). For ITT, mice were fasted overnight, and blood glucose was measured at 0, 30, 60, 90, and 120 min after intraperitoneal injection with human insulin (0.75 unit/kg of body weight, Thermo Fisher Scientific, Waltham, MA, USA). The area under the curve (AUC) for blood glucose was calculated.

### 2.14. Lipid peroxidation

The levels of MDA, a product of lipid peroxidation, in the liver of HFD-fed mice were determined by assay kits according to the manufacturer's instructions.

### 2.15. PLA

PLA was performed as per the manufacturer's instructions. Huh7 cells or liver sections were incubated with Duolink blocking buffer at 37℃ for 1 h. The specific primary rabbit anti-fetuin-A antibody or mouse anti-IR antibody were used and sections were then incubated with or without secondary proximity ligation assay probes against rabbit or mouse IgG at 37℃ for 2 h, followed by incubation with ligation buffer for 30 min at 37℃. The sections then were incubated with amplification for 2 h at 37℃. After DAPI staining, the images of PLA assay were captured by Zeiss LSM 880 confocal microscope with Zen software.

### 2.16. Statistical analysis

The results were presented as the mean ± SEM. One-way ANOVA followed by an LSD test was used for multiple comparisons. The Mann-Whitney U test was used to compare two independent groups. SPSS software v8.0 (SPSS Inc., Chicago, IL, USA) was used for all statistical analyses. Differences were considered statistically significant at *P* <0.05.

## 3. Results

### 3.1. Apigenin decreases PA-induced fetuin-A protein expression and promotes the degradation of fetuin-A via autophagy/lysosome pathway in Huh7 cells

**Figure 1:**
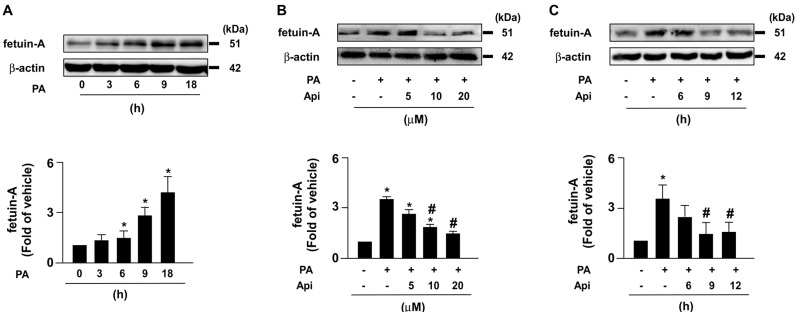
** Apigenin decreases palmitic acid (PA)‐induced expression of fetuin‐A protein in hepatocytes.** (A) Huh7 cells were treated with PA (300 μM) for the indicated times (0, 3, 6, 9, and 18 h). (B) Huh7 cells were pretreated with PA for 6 h, and then incubated with the indicated concentrations (0, 5, 10, and 20 μM) of apigenin (Api) for an additional 12 h. (C) Huh7 cells were pretreated with PA (300 μM) for 6 h, and then with Api (20 μM) for the indicated times (0, 6, 9, and 12 h). Western blot analysis of fetuin-A and β-actin. Data from 5 independent experiments are expressed as mean ± standard error of the mean (SEM). *p < 0.05 vs. the 0 h or the vehicle group. #*p* < 0.05 vs. the PA alone group.

**Figure 2:**
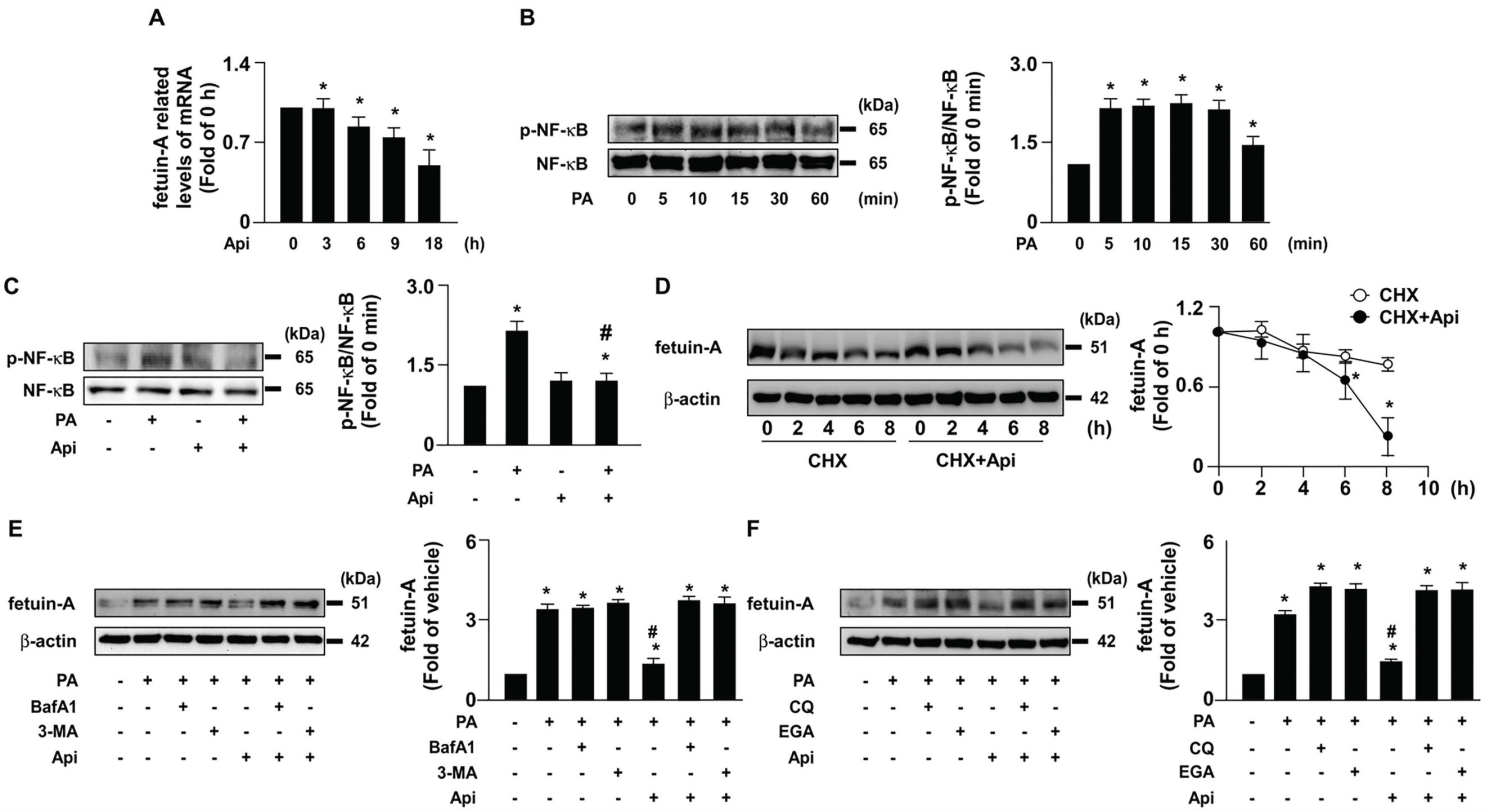
** Apigenin decreases the fetuin-A mRNA expression by decreasing palmitic acid (PA)-induced phosphorylation of nuclear factor kappa B (NF-κB) and promotes the degradation of fetuin‐A protein by activating the autophagy pathway in hepatocytes.** (A) Huh7 cells were pretreated with PA (300 μM) for 6 h, and then incubated with apigenin (Api, 20 μM) for the indicated times (0, 3, 6, 9, and 18 h). The fetuin-A mRNA expression was assessed by real-time PCR analysis. (B) Huh7 cells were treated with PA (300 μM) for the indicated times (0, 5, 10, 15, 30, and 60 min). The levels of NF-κB phosphorylation and total NF-κB were examined by western blot analysis. (C) Huh7 cells were pretreated with Api (20 μM) for 12 h, and then incubated with or without PA for 5 min. The levels of NF-κB phosphorylation and total NF-κB were evaluated by western blot analysis. (D-F) Western blot analysis of fetuin-A and β-actin. (D) Huh7 cells were pretreated with PA (300 μM) for 18 h, and then treated with Api (20 μM) in the presence of cycloheximide (CHX, 20 μg/mL) for the indicated times (0, 2, 4, and 8 h). (E) Huh7 cells were pretreated with PA (300 μM) for 6 h, and then incubated with autophagy inhibitors (bafilomycin A1, BafA1, 100 nM or 3-methyladenine, 3-MA, 5 μM) for 2 h, followed by Api (20 μM) for an additional 12 h. (F) Huh7 cells were pretreated with or without PA (300 μM) for 6 h, and then incubated with lysosome pathway inhibitor (chloroquine, CQ, 40 μM) or late endosomal pathway inhibitor 4-bromobenzaldehyde N-(2,6-dimethylphenyl) semicarbazone (EGA) (10 μM) for 2 h, followed by Api (20 μM) for an additional 12 h. Data from 5 independent experiments are expressed as mean ± standard error of the mean (SEM). *p < 0.05 vs. the vehicle group. #*p* < 0.05 vs. the PA alone group.

To elucidate the potential effects of apigenin on insulin resistance in hepatocytes, we used PA-induced insulin resistance in Huh7 cells as an *in vitro* model. We first studied the cytotoxic effect of apigenin on the cell viability. The exposure of Huh7 cells to PA and various concentrations (0, 5, 10, 20, and 40 μM) of apigenin and the results of the MTT assay showed that apigenin had no cytotoxic effect on cell viability at the concentrations ranging from 5 to 20 μM. However, apigenin induced cell death at the concentration of 40 μM (**Supplementary Fig. S1**). Next, our data showed that fetuin-A levels increased in a time-dependent manner (**Fig. 1A**). We treated Huh7 cells with or without apigenin, and our results revealed that apigenin treatment abolished PA-induced fetuin-A expression in a concentration- and time-dependent manner (**Figs. 1B** and **C**). Subsequently, we examined the molecular mechanisms underlying the inhibitory effects of apigenin on PA-induced fetuin-A upregulation. Apigenin treatment decreased fetuin-A mRNA expression (**Fig. 2A**). Additionally, PA-induced an increase in the phosphorylation levels of NF-κB induction (**Fig. 2B**); however, such an increase was diminished by apigenin treatment (**Fig. 2C**). Moreover, treatment with apigenin promoted fetuin-A degradation in a time-dependent manner (**Fig. 2D**). Pretreatment with BafA1 or 3-MA, two autophagy pathway inhibitors, inhibited the effects of apigenin on fetuin-A degradation (**Fig. 2E**). Pretreatment with CQ and 4EGA, which are lysosomal and late endosomal pathway inhibitors, respectively, also abolished the effect of apigenin on fetuin-A protein degradation (**Fig. 2F**). Collectively, these results suggest that apigenin modulates fetuin-A expression by regulating transcriptional and post-translational pathways.

### 3.2. Apigenin decreases the CK2α-dependent phosphorylation of fetuin-A and improves PA-impaired glucose uptake in Huh7 cells

**Figure 3:**
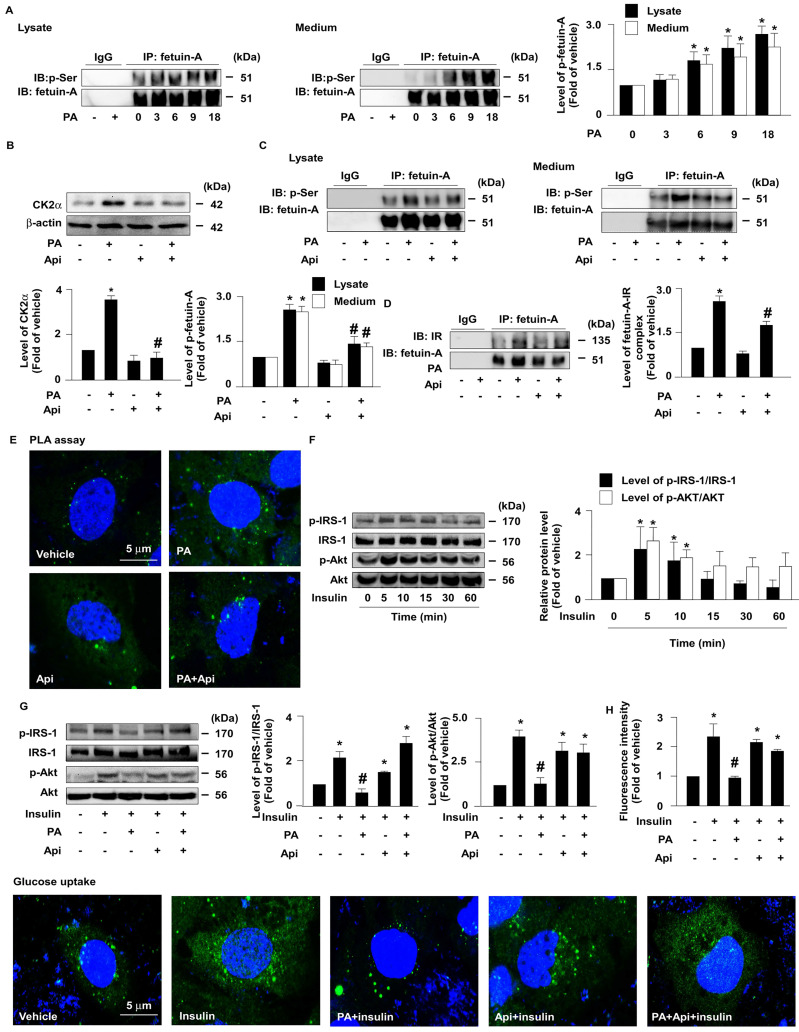
** Apigenin inhibits casein kinase 2α (CK2α)-mediated fetuin-A phosphorylation and prevents the palmitic acid (PA)-induced deregulation of insulin signaling.** (A and B) Huh7 cells were pretreated with PA (300 μM) for the indicated times (0, 3, 6, 9, and 18 h). Cellular lysates or cultured medium were immunoprecipitated (IP) with an anti-fetuin-A antibody and immunoblotting (IB) was performed with an anti-phosphor (p-Ser) antibody. IgG was used as a control for the fetuin-A antibody. (B) Western blot analysis of casein kinase 2α (CK2α) and β-actin. (C and D) Huh7 cells were pretreated with PA (300 μM) for 6 h and then incubated with Api (20 μM) for 12 h. Cellular lysates or cultured medium were IP with anti-fetuin-A antibody and IB was performed with anti-phosphos (p-Ser) antibody. IgG was used as a control for the fetuin-A antibody. (D) Cellular lysates were IP with anti-fetuin-A antibody and IB was then performed with anti-IR or anti-fetulin-A antibody. IgG was used as a control. (E) Representative confocal microscopy images of proximity ligation assay (PLA) performed using anti-fetuin-A and anti-insulin receptor (IR) antibodies. 4',6-diamidino-2-phenylindole (DAPI) (blue), proximity ligation assay (PLA) signals (green). (F and G) Western blot analysis of phosphorylated levels of insulin receptor substrate (IRS) (p-IRS) and Akt (p-Akt), and total levels of IRS and Akt. (F) Huh7 cells were treated with insulin (100 nM) for the indicated times (0, 5, 10, 15, 30, and 60 min). (G) Huh7 cells were pretreated with PA (300 μM) for 6 h and then incubated with Api (20 μM) for 5 min. (H) Cells were pretreated with PA (300 μM) for 6 h and then incubated with Api (20 μM) for 12 h, followed by insulin (100 nM) for 20 min in the presence of 2-deoxy-D-glucose (2-NBDG) (50 μM). Representative images of 2-NBDG (green) and DAPI (blue) by confocal microscopy. Glucose uptake was assessed using the assay kit. Data are expressed as mean ± standard error of the mean (SEM) from 5 independent experiments. *p < 0.05 vs. the vehicle group. #*p* < 0.05 vs. the PA alone group.

Next, we explored whether the apigenin-promoted degradation of fetuin-A was mediated through the regulation of fetuin-A phosphorylation. The results of the co-immunoprecipitation (IP) assay revealed that treatment with PA increased the levels of p-fetuin-A in a time-dependent manner in both the cell lysates and culture media (**Fig. 3A**). Additionally, treatment with apigenin decreased the protein levels of CK2α (**Fig. 3B**) and the phosphorylated levels of fetuin-A (**Fig. 3C**). Fetuin-A binds to IR and interferes with the autophosphorylation of IR and glucose internalization 27-29. Our data further showed that apigenin decreased fetuin-A-IR complex formation (**Fig. 3D** and **E**). Moreover, apigenin rescued the PA-induced deregulation of insulin signaling by preventing the PA-induced decrease in the levels of phosphorylated insulin receptor substrate-1 (IRS-1) and Akt (**Figs. 3F** and **G**), and 2-NBDG uptake by insulin (**Fig. 3H**). Collectively, these findings suggest that apigenin improves insulin resistance by inhibiting CK2α-dependent phosphorylation of fetuin-A and the formation of the fetuin-A-IR complex in hepatocytes.

### 3.3. Apigenin decreases PA-induced intracellular oxidative stress in hepatocytes

**Figure 4:**
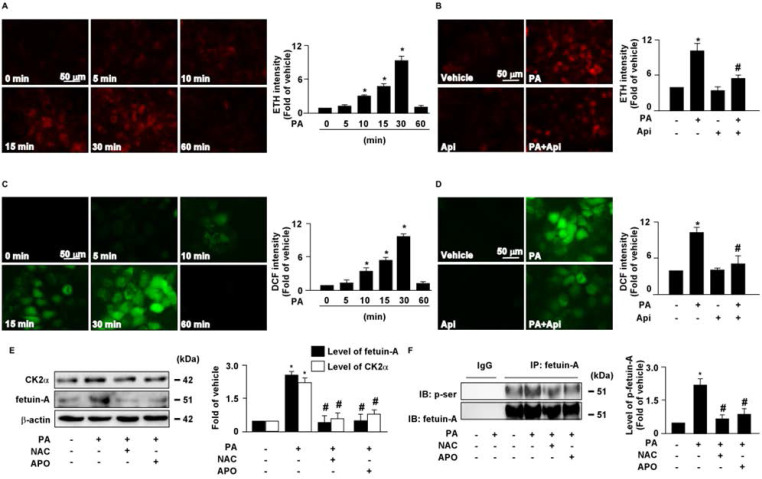
** Apigenin prevents the palmitic acid (PA)‐induced increase in generation of reactive oxygen species in hepatocytes.** (A and B) The intracellular level of superoxide was evaluated with hydroethidine (HE)/ethidium (ETH) fluorescent probe assay. (A) Huh7 cells were treated with PA (300 μM) for indicated times (0, 5, 10, 15, 30, and 60 min). (B) Huh7 cells were pretreated with apigenin (Api, 20 μM) for 2 h, and then with PA (300 μM) for 30 min. (C and D) The intracellular level of hydrogen peroxide was evaluated using dichlorodihydrofluorescein diacetate (DCFH-DA)/2', 7'-dichlorofluorescein (DCF) fluorescent probe assay. (C) Huh7 cells were treated with PA (300 μM) for the indicated times (0, 5, 10, 15, 30, and 60 min). (D) Huh7 cells were pretreated with Api (20 μM) for 2 h, and then with PA (300 μM) for 30 min. (E) Huh7 cells were pretreated with N-acetylcysteine (NAC, 10 mM) or apocynin (Apo, 50 μM) for 2 h, and then incubated with PA (300 μM) for an additional 6 h. Western blot analysis of CK2α, fetuin-A, and β-actin. (F) Huh7 cells were pretreated with NAC (10 mM) or Apo (50 μM) for 2 h, and then incubated with PA (300 μM) for an additional 12 h. Cellular lysates were immunoprecipitated (IP) with an anti-fetuin-A antibody and then immunoblotted with anti-phospho (p-Ser) antibody. IgG was used as a control for fetuin-A antibody. Data are expressed as mean ± standard error of the mean (SEM) from 5 independent experiments. *p < 0.05 vs. the vehicle group. #*p* < 0.05 vs. the PA alone group.

Increased levels of intracellular ROS contribute to the pathogenesis of insulin resistance 30. Our results showed that treatment with PA increased intracellular ROS production in a time-dependent manner (**Figs. 4A** and **C**), which was abolished by apigenin treatment (**Figs. 4B** and **D**). We then examined the effect of ROS on the expression of CK2α and fetuin-A as well as the phosphorylation of fetuin-A. The results revealed that pretreatment with the ROS scavenger NAC and the NOX inhibitor APO abolished the PA-induced increase in the protein levels of CK2α, fetuin-A, and p-fetuin-A (**Figs. 4E** and **F**). These findings suggest that apigenin abrogates the PA-induced increase in the levels of CK2α, fetuin-A, and p-fetuin-A by inhibiting the generation of ROS in hepatocytes.

### 3.4. Apigenin reduces body weight and tissue weights, and reduces the lipids in plasma and the liver in HFD-fed mice

**Figure 5:**
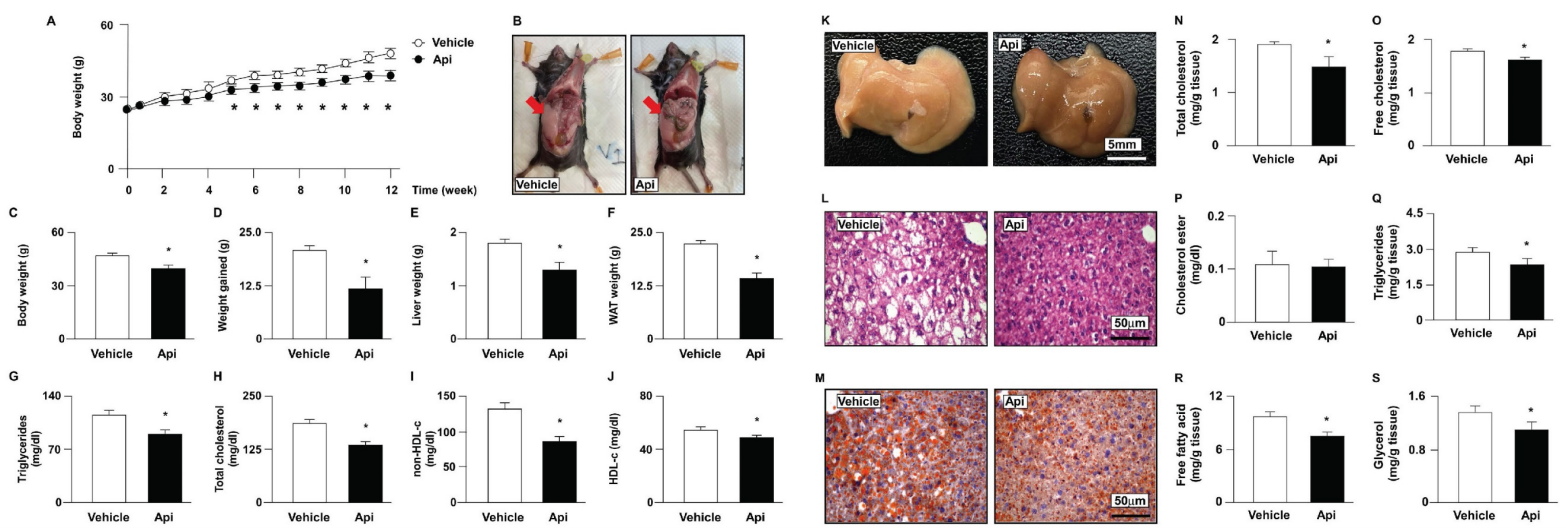
** Effects of apigenin on body weight, tissue weight, and plasma lipids of HFD-fed mice.** Eight-week-old C57BL/6 mice were fed with HFD and orally treated daily with apigenin (Api, 20 mg/kg) or vehicle (oil) for 12 weeks. (A) Weekly body weight changes in vehicle- and apigenin-treated groups. (B) The images of body appearance and the distribution of white adipose tissue (WAT) were indicated by arrows. (C) The body weight. (D) The weight gained. (E) The weight of the liver. (F) The weight of WAT. (G-J) Serum levels of triglycerides, total cholesterol, non-high-density lipoprotein cholesterol (non-HDL-c), and HDL cholesterol (HDL-c). (K-M) The appearance of the liver and representative histological images by hematoxylin and eosin (H&E) staining and Oil Red O staining. (N-S) The hepatic levels of total cholesterol, free cholesterol, cholesteryl ester, triglycerides, free fatty acids, and glycerol. Data are expressed as mean ± standard error of the mean (SEM) from 7 mice. *p < 0.05 vs. the vehicle-treated group.

**Figure 6:**
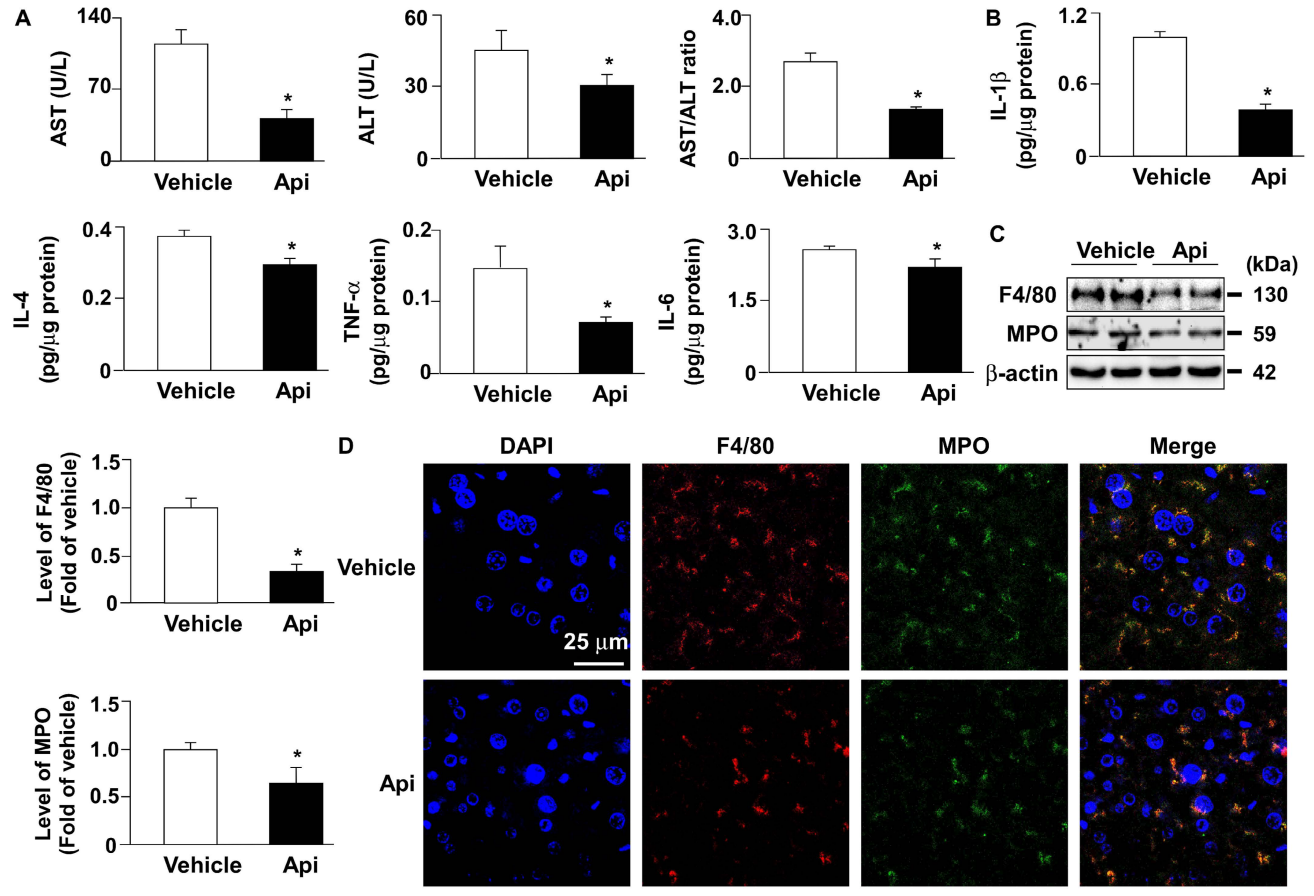
** Apigenin decreases hepatic inflammation in the liver of HFD-fed mice.** Eight-week-old C57BL/6 mice were fed with HFD and orally treated daily with apigenin (Api, 20 mg/kg) or vehicle (oil) for 12 weeks. (A) The serum levels of aspartate aminotransferase (AST), alanine aminotransferase (ALT), and the ratio of AST/ALT. (B) The hepatic levels of interleukin 1β (IL-1β), IL-4, tumor necrosis factor-alpha (TNFα) and IL-6. (C) Western blot analysis of F4/80, myeloperoxidase (MPO), and β-actin. (D) Immunohistochemistry of F4/80 and MPO in the liver sections. Data are expressed as mean ± standard error of the mean (SEM) from 7 mice. *p < 0.05 vs. the vehicle group.

To explore the potential effects of apigenin on obesity-induced insulin resistance, eight-week-old HFD-fed mice were treated with or without apigenin. Our results demonstrated that HFD-fed mice treated daily with apigenin for 12 weeks exhibited lower body weight, liver weight, white adipose tissue (WAT) weight, and plasma lipid levels, including triglycerides, total cholesterol, non- HDL-c, and HDL-c, than vehicle-treated HFD-fed mice (**Figs. 5A-J**). Abnormal lipid accumulation in the liver is linked to insulin resistance 31. We therefore examined the effect of apigenin on lipid levels in the livers of HFD-fed mice. Compared to the vehicle-treated group, treatment with apigenin changed the appearance of the liver (**Fig. 5K**) and decreased lipid accumulation in the liver, as determined by hematoxylin and eosin (H&E) and Oil Red O staining (**Figs. 5L** and **M**). Moreover, treatment with apigenin decreased the levels of total cholesterol, free cholesterol, triglycerides, free fatty acids, and glycerol in the liver of HFD-fed mice (**Figs. 5N-S**). To determine the effect of apigenin on hepatic function and inflammation, we measured the serum levels of AST and ALT to confirm the effect of apigenin on hepatic function. Our results revealed that the serum levels of AST and ALT were significantly decreased in the apigenin-treated HFD-fed mice (**Fig. 6A**). Furthermore, the hepatic levels of inflammatory cytokines IL-1β, IL-4, TNFα and IL-6 were decreased in apigenin-treated HFD-fed mice as compared to that of the vehicle-treated mice (**Fig. 6B**). Moreover, treatment with apigenin decreased the protein levels of MPO and F4/80, the cell markers for neutrophils and macrophages, respectively, in the livers of HFD-fed mice (**Fig. 6C** and** D**). We next investigated the effect of apigenin on WAT in HFD-fed mice. Our results indicated that after apigenin treatment, the weight of WAT was decreased and the expression of lipolysis pathway-related proteins including ATGL, HSL, LAL, and LC3-II was increased; in contrast, the expression of p62 was decreased (**Supplementary Fig. S2**). Together, these results suggest that apigenin exerts a protective effect against HFD-induced obesity and the hepatic deregulation of lipid metabolism, and inflammation.

### 3.5. Apigenin downregulates the expression of fetuin-A in HFD-fed mice

**Figure 7:**
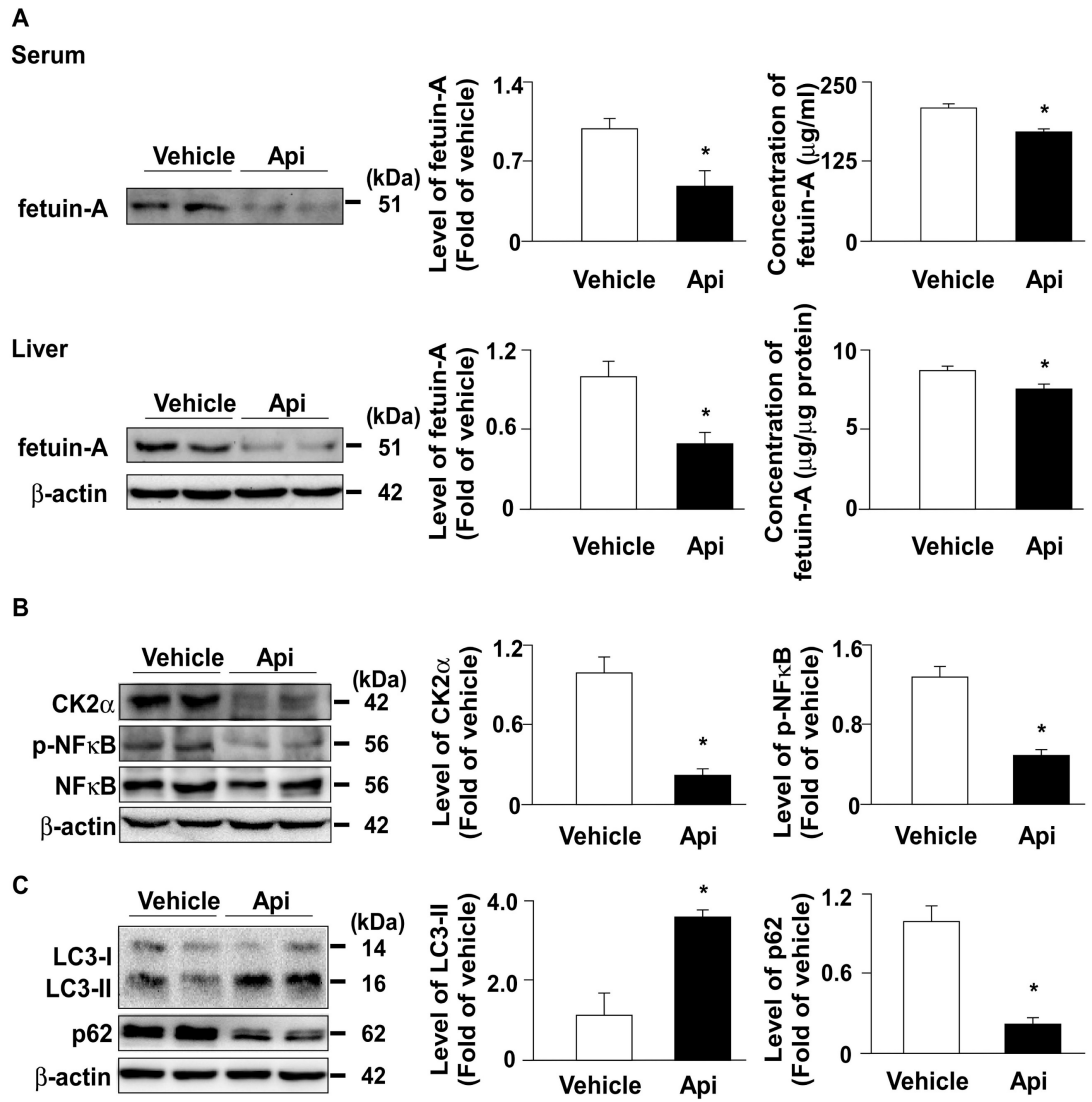
** Apigenin decreases the expression of fetuin-A protein by regulating CK2α -NF-κB and autophagy pathways in the liver of HFD-fed mice.** Eight-week-old C57BL/6 mice were fed with HFD and treated daily with apigenin (Api, 20 mg/kg) or vehicle (oil) for 12 weeks. (A) Western blot analysis and ELISA analysis of the serum and hepatic of fetuin-A. (B and C) Western blot analysis of CK2α, phosphorylated and total NF-κB, LC3, p62, and β-actin in the liver. Data are expressed as mean ± standard error of the mean (SEM) from 7 mice. *p < 0.05 vs. the vehicle-treated group.

To confirm the *in vitro* observations, we investigated the effect of apigenin on the expression of fetuin-A and related regulatory mechanisms in HFD-fed mice. Our results showed that administration with apigenin decreased the levels of fetuin-A protein both in the serum and the liver of HFD-fed mice (**Fig. 7A**). Furthermore, the levels of CK2α and p-NF-κB protein were decreased in the liver upon apigenin administration (**Fig. 7B**). Moreover, the autophagy marker LC3 increased and p62 decreased, suggesting that apigenin activates the autophagy-lysosome pathway (**Fig. 7C**). These results suggest that apigenin decreases fetuin-A protein expression by regulating CK2α-NF-κB-dependent transcriptional signaling and autophagy-lysosome-dependent post-translational pathway.

### 3.6. Apigenin decreases oxidative stress by increasing the protein expression of antioxidant detoxification system in the liver of HFD-fed mice

**Figure 8:**
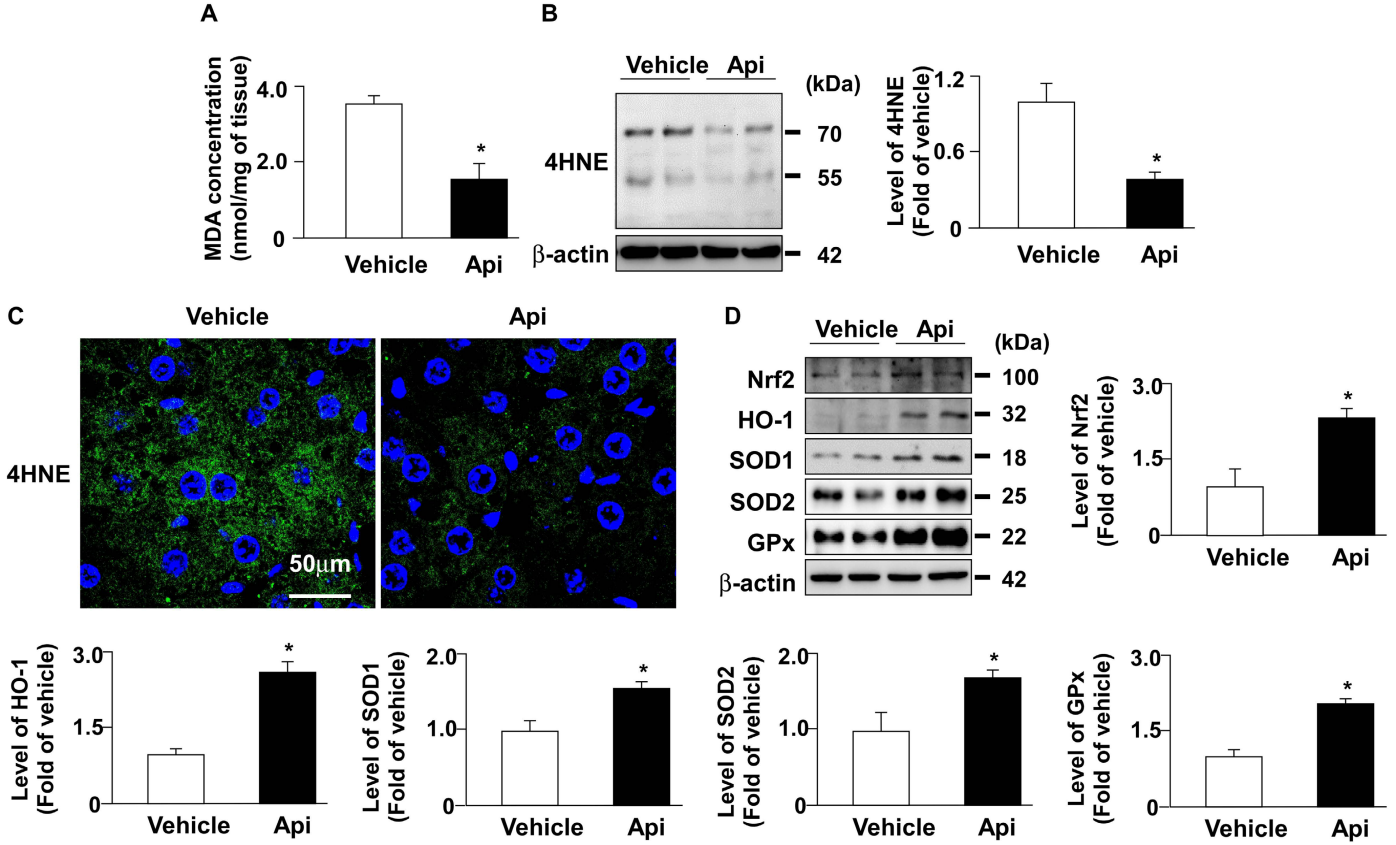
** Apigenin reduces oxidative stress in the liver of HFD-fed mice.** Eight-week-old C57BL/6 mice were fed with HFD and treated daily with apigenin (Api, 20 mg/kg) or vehicle (oil) for 12 weeks. (A) The level of lipid peroxidation in the liver. (B) Western blot analysis of 4-hydroxynonenal (4-HNE) and β-actin. (C) Immunohistochemistry of 4-HNE in the liver sections. (D) Western blot analysis of nuclear factor erythroid 2-related factor 2 (Nrf2), heme oxygenase-1 (HO-1), superoxide dismutase (SOD)1, SOD2, glutathione peroxidase (GPx), and β-actin in the liver. Data are expressed as mean ± standard error of the mean (SEM) from 7 mice. *p < 0.05 vs. the vehicle group.

Hepatic ROS plays an important role in the pathogenesis of obesity-induced insulin resistance 32. Next, we investigated whether apigenin decreased oxidative stress and its potential mechanisms in the livers of HFD-fed mice. As shown in **Fig. 8**, the administration of apigenin decreased the levels of lipid peroxidation and 4-HNE in the liver of HFD-fed mice (**Figs. 8A-C**). Moreover, apigenin increased the protein levels of Nrf2, HO-1, GPx, and SOD2 in the liver of HFD-fed mice (**Fig. 8D**). These findings suggest that apigenin reduces the HFD-induced increase in oxidative stress by upregulating the expression of proteins in the antioxidant detoxification system.

### 3.7. Apigenin improves insulin resistance in the liver of HFD-fed mice

**Figure 9:**
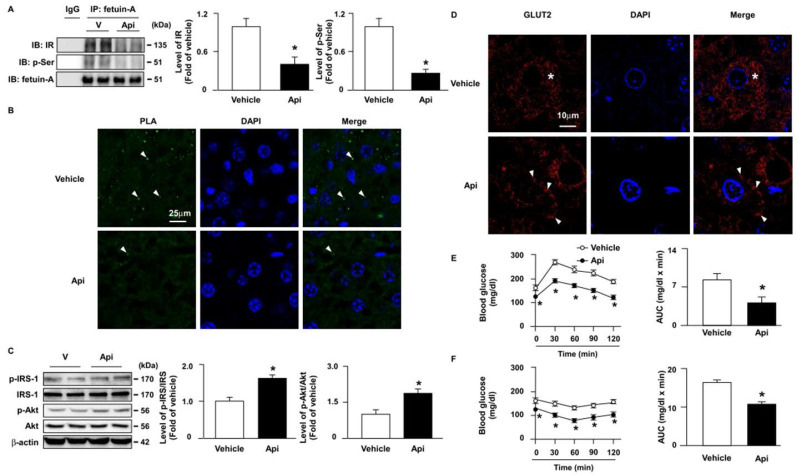
** Apigenin attenuates HFD-induced insulin resistance by inhibiting fetuin-A phosphorylation and the formation of the fetuin-A-insulin receptor complex in the liver of mice.** Eight-week-old C57BL/6 mice were fed with HFD and treated daily with apigenin (Api, 20 mg/kg) or vehicle (oil) for 12 weeks. (A) Liver lysates were immunoprecipitated (IP) with an anti-fetuin-A antibody, and immunoblotting (IB) was performed with anti-insulin receptor (IR) or anti-p-Ser antibodies. IgG was used as a control. (B) Representative confocal microscopy images of proximity ligation assay (PLA) were performed using anti-fetuin-A and anti-IR antibodies. The formation of fetuin-A-IR in liver sections was indicated by arrows. 4',6-diamidino-2-phenylindole (DAPI) (blue), PLA signals (green). (C) Western blot analysis of phosphorylated and total and of the IR substrate (IRS) and Akt. (D) Representative confocal microscopy images of glucose transporter 2 (GLUT2) in liver sections; GLUT2 located in cytosol is indicated by stars, GLUT2 located around cell membrane is indicated by arrows. (E) Results of oral glucose tolerance test (OGTT). Mice were orally administrated with glucose (1.0 g/kg of body weight) and the levels of blood glucose were measured at the indicated time points (0, 30, 60, 90, and 120 min). The area under curve (AUC) for OGTT was calculated. (F) Results of insulin tolerance test (ITT). Mice were intraperitoneally injected with insulin (0.75 unit/kg of body weight) and the levels of blood glucose were measured at the indicated time points (0, 30, 60, 90, and 120 min). AUC for ITT was calculated. Data are expressed as mean ± standard error of the mean (SEM) from 7 mice. *p < 0.05 vs. the vehicle-treated group.

Next, we explored the effect of apigenin on insulin resistance and its underlying mechanisms. Our results showed that the administration of apigenin decreased the formation of the fetuin-A-IR complex (**Figs. 9A** and **B**) and the phosphorylation levels of fetuin-A (**Fig. 9A**) in the liver of HFD-fed mice. Compared with vehicle-treated HFD-fed mice, the phosphorylated levels of insulin signaling pathway-related proteins, including IRS and Akt, were increased in the liver of apigenin-treated HFD-fed mice (**Fig. 9C**). Additionally, GLUT2 was translocated from the cytosol to the plasma membrane in the hepatocytes of apigenin-treated HFD-fed mice (**Fig. 9D**). Moreover, the results of the OGTT and ITT showed that apigenin decreased the plasma levels of glucose and improved insulin resistance in HFD-fed mice (**Figs. 9E** and **F**). Taken together, these results suggest that apigenin improves insulin resistance and glucose uptake by downregulating fetuin-A phosphorylation and its interactions with IR.

## 4. Discussion

**Figure 10:**
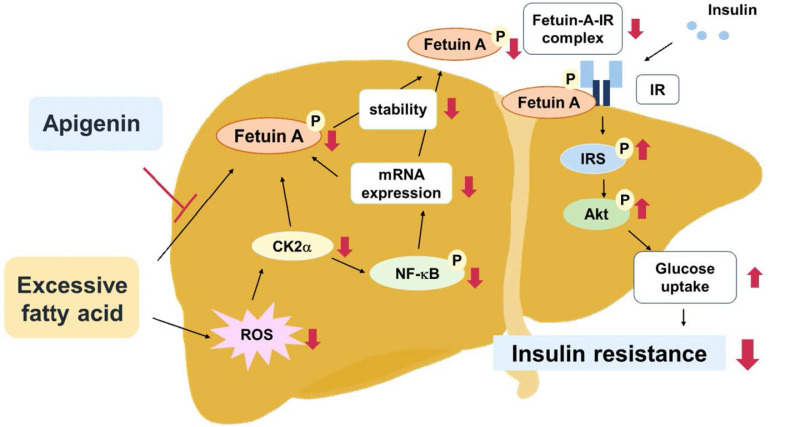
** Schematic illustration of the proposed molecular mechanisms by which apigenin protects the liver from obesity-induced insulin resistance.** As shown, treatment with apigenin prevents lipid-induced deregulation of glucose metabolism by inhibiting ROS-induced CK2α expression and CK2α-mediated activation of signaling pathways including the phosphorylation of NF-κB and fetuin-A, resulting in reduction in the expression and stability of fetuin-A protein thereby preventing the formation of fetuin-A-insulin receptor (IR) complex, and ultimately improving obesity-induced insulin resistance.

Apigenin exerts protective effects against obesity, diabetes, and its related complications 22,26. Fetuin-A binds to IR and interferes with the activation of the insulin signaling pathway; therefore, it plays a key role in the pathogenesis of insulin resistance and type 2 diabetes mellitus (T2DM) 14,15. However, the interlocked biology of apigenin and fetuin-A has not yet been investigated. In this study, we characterized a new mechanism by which fetuin-A downregulation contributes to the beneficial effects of apigenin on obesity-induced dysregulation of insulin signaling in hepatocytes and the liver. In this study, PA-induced and HFD-induced insulin resistance were used as *in vitro* and *in vivo* models, respectively, to explore the potential molecular mechanisms underlying the beneficial effects of apigenin on insulin resistance. Our results demonstrate that exposing hepatocytes to excess PA rapidly increased the generation of ROS and induced the activation of CK2α-NF-κB pathway, which in turn upregulated the fetuin-A mRNA expression. In addition, PA-activated CK2α caused an increase in the phosphorylated form of fetuin-A protein, thereby increasing its half-life. Both transcriptional and posttranslational regulation may work in concert to upregulate fetuin-A protein levels in hepatocytes. Moreover, apigenin abolished the PA-induced increase in fetuin-A protein levels by inhibiting ROS generation and CK2α-dependent transcriptional and posttranslational regulation. Consequently, apigenin prevented the PA-induced increase in fetuin-A expression and formation of the fetuin-A-IR complex, and thus rescued the PA-induced impairment of insulin signaling and glucose uptake in hepatocytes. Similar results were observed in HFD-fed mice, in which apigenin decreased oxidative stress in the liver, prevented the HFD-induced increase in circulating levels of fetuin A, and ultimately protected the mice from hyperglycemia and insulin resistance (**Fig. 10**). The findings of this study provide new insights into the therapeutic effects of apigenin and its regulatory mechanisms on insulin resistance and T2DM.

Fetuin-A is a multifunctional protein correlated with insulin resistance 33-35. Notably, our findings regarding the inhibitory effect of apigenin on the regulation of fetuin-A protein expression were consistent with those of Mathews et al., who found that genetic deletion of fetuin-A improved insulin sensitivity and resistance in obese mice 34,36. Heo et al. reported that melatonin improved hepatic insulin resistance and steatosis by downregulating the expression of fetuin-A 37. Moreover, a clinical trial by Ghadimi et al. showed that ellagic acid supplementation decreased inflammation and insulin resistance in diabetic patients 38. Mechanistically, our results revealed that apigenin downregulated fetuin-A mRNA expression by decreasing the activation of NF-κB, which is a key transcriptional factor involved in the regulation of immune response and insulin resistance 39,40. Our observations were consistent with the findings of Dasgupta et al., who reported that NF-κB activation is required for PA-induced fetuin-A expression in hepatocytes 39. Collectively, these lines of evidence strongly suggest that fetuin-A is a potential therapeutic target for the treatment of insulin resistance and related metabolic diseases.

Apart from the transcriptional regulation by NF-κB, posttranslational modification such as CK2α-dependent phosphorylation at Ser120 and Ser312 of fetuin-A has been reported to be an important regulatory mechanism for increasing the protein stability of circulating fetuin A 5,14. Ren et al. reported that the phosphorylation status of fetuin-A is highly associated with obesity and insulin resistance 15. Particularly, the phosphorylation of fetuin-A at Ser312 plays a critical role in the inhibition of the insulin signaling pathway 15. However, there is no commercial antibody targeting the phosphorylated Ser312 of fetuin-A, and we therefore performed immunoprecipitation using anti-fetuin-A and immunoblotting (IB) using a phospho-Ser antibody to examine the effect of apigenin on the PA- or HFD-induced phosphorylation of fetuin-A. Our results demonstrated that apigenin decreased the phospho-Ser levels of fetuin-A induced by PA or HFD. Interestingly, apigenin is known to be a CK2α inhibitor that contributes to cancer therapy 13,41, which is in line with our findings that apigenin decreases the expression of CK2α resulting in a decrease in the levels of p-fetuin-A. This was confirmed by the observation that treatment with CX4945, another CX2α inhibitor, prevented PA-induced fetuin-A phosphorylation (**Supplementary Fig. S3**). Taken together, these findings suggest that apigenin exerts beneficial effects by regulating fetuin-A function through both the transcriptional and posttranslational pathways. However, the detailed molecular mechanism by which apigenin regulates the activation of CK2α and NF-κB in the hepatocytes warrants further investigation.

In addition to its adverse effects on insulin resistance, fetuin-A has been correlated with other metabolic diseases 42,43. von Loeffelholz et al. showed that compared to patients with non-nonalcoholic fatty liver disease (non-NAFLD), circulating levels of fetuin-A were increased in patients with NAFLD, and Naito et al. reported that fetuin-A induced endothelial dysfunction and promoted macrophage foam cell formation, leading to the progression of atherosclerosis 42,43. Apigenin has been reported to be beneficial for the treatment of metabolic diseases, including obesity, NAFLD, and atherosclerosis 22,44. However, whether the inhibition of fetuin-A contributes to apigenin-mediated protection from the above-mentioned metabolic diseases remains unclear. Further investigations are required to explore the connection between fetuin-A and the protective effects of apigenin on lipid disorders.

ROS play an important role in regulating cellular physiological functions and pathogenesis of metabolic diseases 45. An imbalance between ROS generation and the detoxifying capacity of antioxidant defense systems increases intracellular oxidative stress, which damages the functions of proteins, lipids, and cellular DNA 25,46. Feng et al. reported that apigenin decreases oxidative stress and attenuates obesity-induced metabolic disorders 25. This notion is further supported by our observation that apigenin inhibited the PA-induced increase in the production of ROS in Huh7 cells. Furthermore, the ROS scavenger NAC and NOX inhibitor Apo abolished PA-induced ROS production and fetuin-A upregulation and phosphorylation, suggesting an essential role of ROS in regulating the function of fetuin-A in the deregulation of hepatic lipid metabolism. Moreover, the administration of apigenin attenuated HFD-induced oxidative stress by increasing the protein expression of antioxidants, including Nrf2, HO-1, SOD1, SOD2, and GPx in the liver. These findings suggest that the antioxidant properties of apigenin may play a crucial role in its beneficial effects against metabolic diseases.

In conclusion, this study demonstrates the unique protective mechanisms of apigenin in treating hepatic insulin resistance, which involves the regulation of fetuin-A gene expression and protein phosphorylation of fetuin-A, and the interaction between fetuin-A and IR, which ultimately leads to the alleviation of obesity-induced insulin resistance in hepatocytes and the liver. Here, we provide new insights into the beneficial effects of apigenin in improving hepatic insulin resistance, broadening the biomedical implications of apigenin for the prevention and treatment of obesity-induced metabolic disorders.

## Supplementary Material

Supplementary figures.

## Abbreviations

2-NBDG: 2-deoxy-2-[(7-nitro-2,1,3-benzoxadiazol-4-yl) amino]-D-glucose
3-MA: 3-methyladenine
4-HNE: 4-hydroxy-2-nonenal
Akt: protein kinase B
APO: apocynin
ATGL: adipose triglyceride lipase
BafA1: bafilomycin A1
CK2α: casein kinase 2α
CQ: chloroquine
DCFH-DA: 2′,7′-dichlorofluorescin diacetate
DHE: dihydroethidine
EGA: 2-[(4-bromophenyl) methylene]-N-(2,6-dimethylphenyl)-hydrazinecarboxamide]
GLUT2: glucose transporter 2
GPx: glutathione peroxidase
HFD: high-fat diet
HO-1: heme oxygenase-1
HSL: hormone-sensitive lipase
IP: immunoprecipitation
IR: insulin receptor
IRS: insulin receptor substrate
ITT: insulin tolerance tests
IL-6: interleukin 6
LC3: microtubule-associated protein 1A/1B-light chain 3
LAL: lysosomal acid lipase
MDA: malondialdehyde
MPO: myeloperoxidase
MTT: 3-(4,5-Dimethylthiazol-2-yl)-2,5-diphenyltetrazolium bromide
NAC: N-acetylcysteine
NF-κB: nuclear factor kappa-light-chain-enhancer of activated B
NRF2: nuclear factor erythroid 2-related factor 2
OGTT: oral glucose tolerance tests
PA: palmitic acid
PLA: proximity ligation assay
ROS: reactive oxygen sepsis
SOD: superoxide dismutase
T2DM: type 2 diabetes mellitus
TNFα: tumor necrosis factor alpha
WAT: white adipose tissue
