# Supplementary material for: Apigenin targets fetuin-A to ameliorate obesity-induced insulin resistance
Source: Int J Biol Sci. 2024 Feb 11;20(5):1563–77. doi: 10.7150/ijbs.91695 (PMC10929183; doi:10.7150/ijbs.91695)
Supplement: Supplementary file 1 — Supplementary figures. [file ijbsv20p1563s1.pdf]

Supplementary Material

Apigenin targets fetuin-A to ameliorate obesity-induced insulin resistance

Man-Chen Hsu, Chia-Hui Chen, Mu-Chun Wang, Po-An Hu, Bei-Chia Guo, Ru-Wen Chang, Chih-Hsien Wang, and Tzong-Shyuan Lee

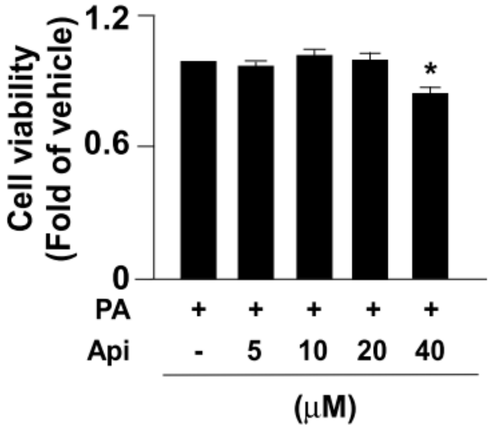

Figure S1. **Effect of apigenin on the cell viability of Huh7 cells.** Huh7 cells were pretreated with PA for 6 h and then incubated with the indicated concentrations of apigenin (Api) (0, 5, 10, 20, and 40 μM) for an additional 12 h. The cell viability was determined by MTT assay. Data from 5 independent experiments are expressed as mean ± standard error of the mean (SEM). *p < 0.05 vs. the PA alone group.

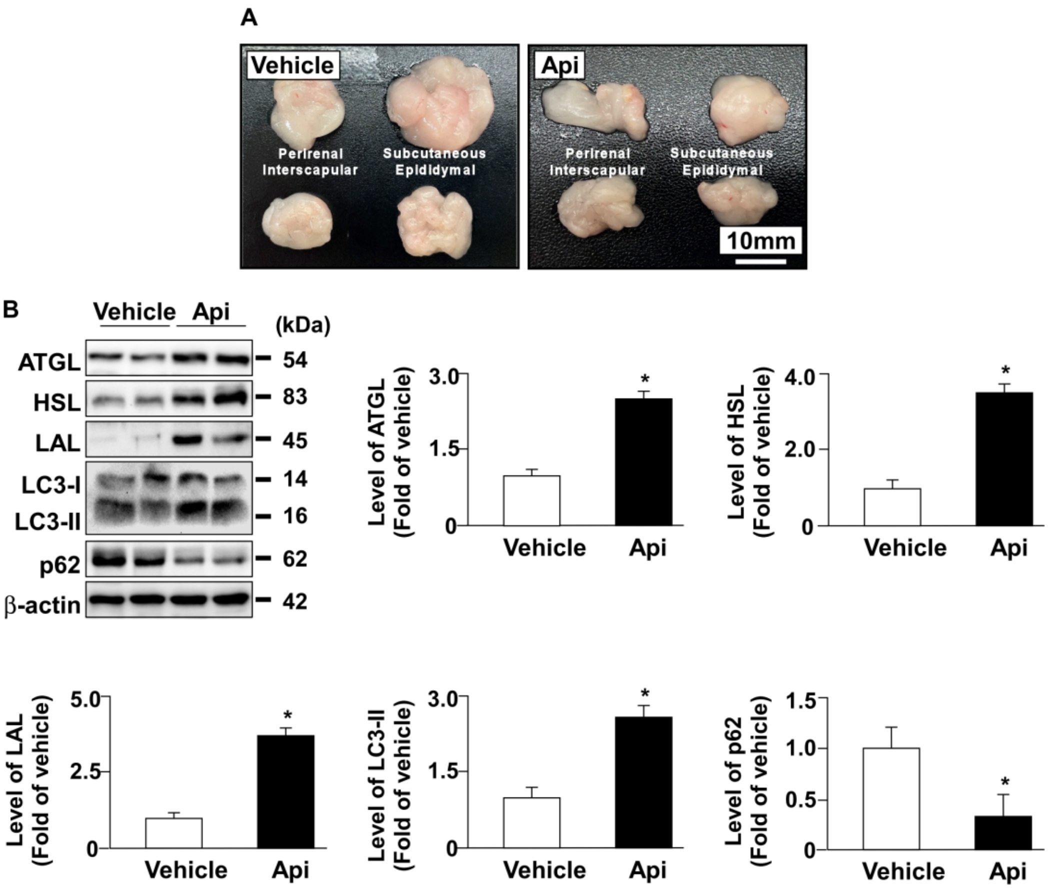

Figure S2. **Apigenin induces lipolysis and lipophagy in WAT of HFD-fed mice.** Eight-week-old C57BL/6 mice were fed with HFD and orally treated daily with apigenin (Api, 20 mg/kg) or vehicle (oil) for 12 weeks. (A) The images of white adipose tissue (WAT) (B) Western blot analysis of adipose triglyceride lipase (ATGL), hormone-sensitive lipase (HSL), lysosomal acid lipase (LAL), LC3, p62 and β-actin in WAT. Data are expressed as mean ± standard error of the mean (SEM) from 7 mice. *$p < 0.05$ vs. the vehicle group.

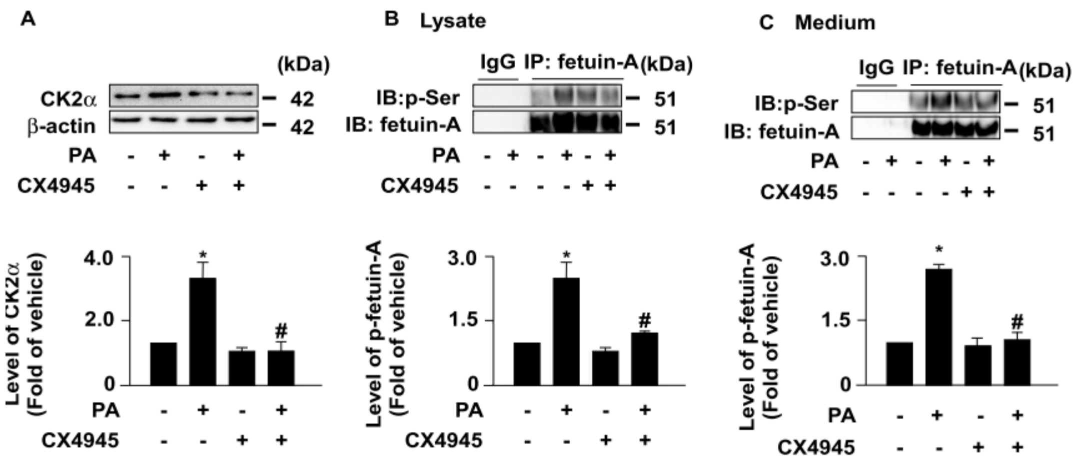

Figure S3. **CX4945 inhibits casein kinase 2α (CK2α)-mediated fetuin-A phosphorylation in Huh7 cells.** Huh7 cells were pretreated with PA (300 μM) for 6 h, and then incubated with CX4945 (5 μM) for 12 h. (A) Western blot analysis of CK2α and β-actin. (B and C) Cellular lysates or cultured medium were immunoprecipitated (IP) with an anti-fetuin-A antibody and immunoblotting (IB) was performed with an anti-phosphor (p-Ser) antibody. IgG was used as a control for the fetuin-A antibody. Data are expressed as mean ± standard error of the mean (SEM) from 5 independent experiments. *$p < 0.05$ vs. the vehicle group. #$p < 0.05$ vs. the PA alone group.
